# A global approach to describe retinal defocus patterns

**DOI:** 10.1371/journal.pone.0213574

**Published:** 2019-04-02

**Authors:** Miguel García García, Dibyendu Pusti, Siegfried Wahl, Arne Ohlendorf

**Affiliations:** 1 Institute for Ophthalmic Research, Eberhard Karls University Tuebingen, Tuebingen, Baden-Wuerttemberg, Germany; 2 Carl Zeiss Vision International GmbH, Aalen, Baden-Wuerttemberg, Germany; 3 Laboratorio de Óptica, Universidad de Murcia, Murcia, Spain; National Eye Institute, UNITED STATES

## Abstract

The popularity of myopia treatments based on the peripheral defocus theory has risen. So far, little evidence has emerged around the questions which of these treatments are effective and why. In order to establish a framework that enables clinicians and researchers to acknowledge the possible interactions of different defocus patterns across the retina, different peripheral refractive errors (PRX) of subjects and different designs of optical treatments were evaluated. Dioptric defocus patterns on the retinal level have been obtained by merging the matrices of dioptric defocus maps of the visual field of different scenarios with individual peripheral refractive errors and different optical designs of multifocal contact lenses. The newly obtained matrices were statistically compared using a non-parametric test with familywise error algorithms and multi-comparison tests. Results show that asymmetric peripheral refractive error profiles (temporal or nasal positively skewed) appear to be less prone to be changed by the defocus imposition of multifocal contact lenses than those presenting symmetric patterns (relative peripheral myopia or hyperopia).

## Introduction

Near-sightedness or myopia is being acknowledged as the new pandemic of the last half-century [1], especially in the face of increasing prevalence, as well as the health and economic burden of this condition. Taking into account the various factors that can influence the refractive development [2], visual feedback is considered as one of the main culprits in the refractive development and the onset and progression of myopia. A widely accepted theory suggests that the eye can detect the sign and amount of defocus and respond on its behalf [3]. Thereby, the sign of defocus would determine the development of refractive errors as has been seen in several animal models [4].

Furthermore, recent studies pointed out that the retina ‘per se’ recognizes local areas that are defocused in order to regulate the global and local growth of the eye [5]. The pathway leading from the optical defocus to the anatomical growth of the eye has not yet been fully described, while studies trend to describe which signals and biochemical binds may result in eye growth [6], the physiological step of how defocus is detected, has remained elusive. A plausible reason for not finding such a step can be that the blur signal has not been yet properly characterised. In general, the blur or defocus signal is generally defined as the mismatch between the focal plane and the receiving (retinal) plane. Homogeneous defocus has been proven to change the growth of the eye in animals, causing myopia under negative lens and hyperopia through positive lenses. However, there is controversy about whether it can promote the same effect in humans, where, for example, under-correction showed an effect for some, but not all, studies [7–10]. Heterogeneous or peripheral blur refers to the situation when different amounts of blur reach the fovea, compared to the periphery of the eye. The relationship between this peripheral refraction and the development of refractive errors is not entirely understood, but substantial evidence links the state of focus outside the fovea with refractive errors [11].

Nonetheless, myopic defocus in the periphery has become one of the strongest trends to slow the progression of myopia [12,13]. Several optical treatments have emerged based on this, such as spectacles with specific designs [14,15], orthokeratology [16] or contact lenses with multifocal designs [17]. These treatments can modify the defocus that reaches the peripheral retina to induce myopic defocus. Besides, evidence has been obtained on whether or not these optical treatments delay the progression of myopia [18], not much has been unveiled about how the relative peripheral myopic defocus translates into a reduction of the progression of myopia. The question remains, as to whether peripheral defocus imposed by those optical treatments is significant or meaningful in the same terms for all the subjects during the progression of myopia, especially when dioptric defocus patterns of real-life scenarios (such as reading, studying or watching TV) are taken into account.

The present study aims to evaluate the significance of different optical treatments, under different environments and different types of peripheral refraction. In addition to this objective, this study also aims to analyse the influence of different types of peripheral refractive errors to improve our understanding around how defocus is distributed in the retina and to create a framework that allows others to study how different optical treatments may behave in the control of myopia.

## Methodology

In order to improve the knowledge about how the level of defocus is distributed across the entire retina and how it changes after the imposition of relative peripheral defocus, the dioptric defocus maps (DDM) of different indoor scenarios, and the peripheral refractive errors maps with and without different optical designs of multifocal contact lenses were measured and combined.

Thirteen myopic students from the Optometry Faculty of the University of Murcia participated in the current study. On average, the subjects had a spherical equivalent error (SE) of -3.25 Dioptres (-0.75 to -6.50 Dioptres) and an axial length of 25.37 mm (22.55 to 26.50 mm). The mean age of the subjects was 26 years (SD ± 6 years). The ethics authorisation to perform the measurements was granted by the Research Ethics Committee from the University of Murcia. ID: 1108/2015. The study adhered to the principles of the Helsinki declaration (1998) and posterior amends and written informed consent was obtained from the subjects, after explaining the study in detail.

The objective refraction of the right eye (OD) was obtained with a Hartmann-Shack sensor (VAO device, AOVIS-1, VOptica SL, Murcia, Spain) [19] and the same eye was subjectively refracted with the same device by an optometrist (author MGG). The biometric measurements including axial length were obtained using a standard optical biometer (Lenstar LS900; Haag-Streit AG, Köniz, Switzerland). Inclusion criteria were as follows: (1) spherical equivalent refractive errors below -7 dioptres, (2) experience in wearing contact lenses, (3) no history of ocular or medical pathology, and (4) without lens opacities.

### Environment maps

The dioptric defocus maps from different indoor environments were taken from a previous study by Garcia et al. [20], where the information of the relative distance between the gaze point ("accommodative status") and the environmental objects distribution, for a given ±20° around the fovea (total: 40°), were recorded using an RGB-D camera and a head-mounted eye-tracker. For the current work, the maps were flipped upside down and from right to left, in order to match the visual fields between the scenarios and the peripheral refraction. The chosen scenes for the study corresponds with an office, living room and hallway activities.

### Peripheral refraction shell

The peripheral refraction of right eyes was measured using an open-view peripheral wave-front sensor (Voptica SL, Murcia, Spain) as described by Jaeken et al. [21], while the subjects were fixating a laser target in 3 meters distance. The instrument scans over a wide range of horizontal arc (80°) resulting in 81 high-resolution Hartmann-Shack (HS) images in 1.8 seconds. The obtained images are processed up to the eighth order of Zernike polynomials with a 3mm pupil diameter, rendering full aberrometry data for every point by using the software provided by the manufacturer. Although full data of high and low order aberrations is given, only the mean of the SE (M) was considered for the current analysis.

In this study, rather than measuring only the mean horizontal meridian or equator, the measurements were performed in 5 parallels or horizontal meridians, asking the subject to fixate at 0°, +10°, +20°, -10° and -20 degrees. Four scans (equivalent to 324 HS images) in each vertical fixation point were averaged to define the peripheral refraction profiles. The threshold of acceptance on each angle was set to 1 Diopter of standard deviation (SD). Points were considered unreliable and therefore removed if the standard deviation out of the four repeated scans was higher than this threshold. All measurements were obtained under natural ocular conditions and without the use of a cycloplegic agent.

### Refractive profile classification

Equatorial (parallel 0°) measurements were used to classify the subject's peripheral refractive profiles into four groups under naked eye conditions, based on the nasal and temporal outer 20 degrees edges compared to the mean refraction in a central area of 20 degrees. The profiles were classified as nasal positively skewed (NPS; n = 5), relative peripheral myopia (RPM; n = 4), relative peripheral hyperopia (RPH; n = 3) and temporal positive skewed (TPS; n = 1).

In the case of NPS, the refractive error at external nasal 20° was more positive than the central and temporal mean refractive errors. If the eye showed relative peripheral myopia, it was classified as RPM, and in case that refraction is more hyperopic in the outer means than in the central, it was noted as RPH. TPS described a peripheral refractive error, where the temporal edge was positively skewed.

Individually, the mean values at each meridian were used to interpolate the rest of the points of a surface fit covering a range of 40° x 40° (resulting in a matrix/map of 400 x 400 pixels with dioptric values at every 0.1 degrees). This dimension was reported to be within the limits at which functional analysis of spot patterns can be carried out [22]. The values of those maps were inverted, so they reflected the power rather than the refraction.

### Contact lens profiles

Two different optical designs of multifocal soft contact lenses (near-centre and far-centre distance) were tested, both with a central error of -0.25D and high addition powers. The contact lens used in the study were the Xtensa, Filcon IV 1 55%, by Mark´ennovy, specifications of the contact lens used can be found in Table 1 [23].

**Table 1:** Contact lens specifications.

Contact Lens	Near-centre	Distance-centre
Brand (Manufacturer)	Xtensa (mark´ennovy)	
Material	Hydrogel Filcon IV 1, 55%	
Dk	19	
Diameter/Base curve	14.30 mm/ 8.60 mm	
Refraction	-0.25 D /—@—Add. High (2.25 D)*	-0.25 D /—@—Add. High (2.50 D)*

*The manufacturer defines the addition power by an approximation to -3.00 Dioptres contact lens profiles.

As in the measurements of the peripheral refraction of the naked eye, the same five horizontal meridians were recorded with the peripheral wave-front sensor (Voptica SL, Murcia, Spain), but in this case, subjects were wearing the contact lenses.

Contact lenses profiles were obtained by performing an 'over-refraction' of the eye while the subject was wearing each of the contact lenses. So, the peripheral refractions from the naked eye condition were subtracted from the on-wear measurements to gain the power profiles of the lenses, as described by Rosén et al. [22]. Measurements of peripheral aberrometry of the naked eye and while wearing two different multifocal contact lens designs (near-centre and distance-centre) were performed in a random order.

As the purpose of the study was to be able to combine information from different conditions that may affect retinal defocus patterns, the mean power profile values in each parallel were also used to interpolate a 40° x 40° surface fit and obtain power profile maps for each optical design. In the case of the naked eye, a blank matrix was used.

### Segmentation & analysis of maps

Thirty-six new maps were computed based on stacked values from the matrices of the 10 possible conditions defined in the study, three different environments (office, corridor and living room), three optical solutions (multifocal contact lens with near-centre or centre-distance design as well as the naked eye condition) and four types of peripheral refraction.

**Fig 1:**
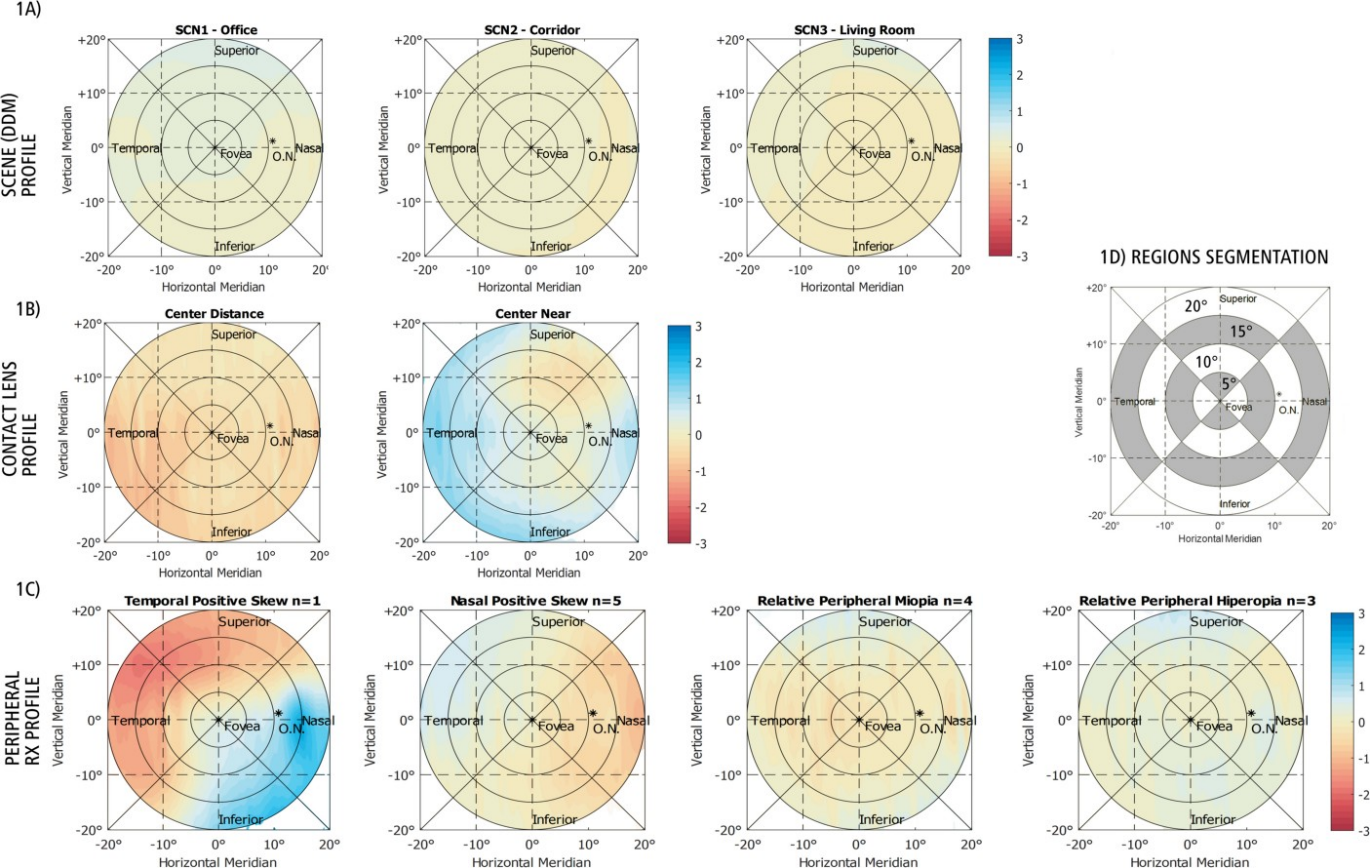
This figure shows the different maps combined with their scale in Dioptres. 1A) Environmental maps. 1B) Contact lens profiles. 1C) Different types of peripheral refractive errors (as power distributions). 1D) Blank map with the segmentation assessment (for display purposes).

The matrices/maps resulting from the combination of the different variables were segmented from inside to the outside diameter (splitting into rings comprehending every 5°). They were also subdivided by retinal coordinates: upper, lower, temporal and nasal segments, resulting in a more detailed assessment. The detailed segmentation, as well as the maps from the different described variables that were used, are shown in Fig 1.

For the analysis, all values from each region under all the possible conditions (scene, optical treatment and peripheral refraction profile) were analysed using the non-parametric Kruskal-Wallis test with family-wise errors (FWER) as a post-hoc analysis. The inner regions up to five degrees contained on average 1961 values while the outer regions (from fifteen to twenty degrees) contained an average of 13737 values.

The data recorded from the subjects was pseudo-anonymised and post-processed within Matlab 2017b (The MathWorks Inc., Natick, MA, USA). All the personal data was stored in accordance with the provisions of the data protection law of the European Union GDPR 2016/679 [24].

## Results

### Baseline conditions

**Fig 2:**
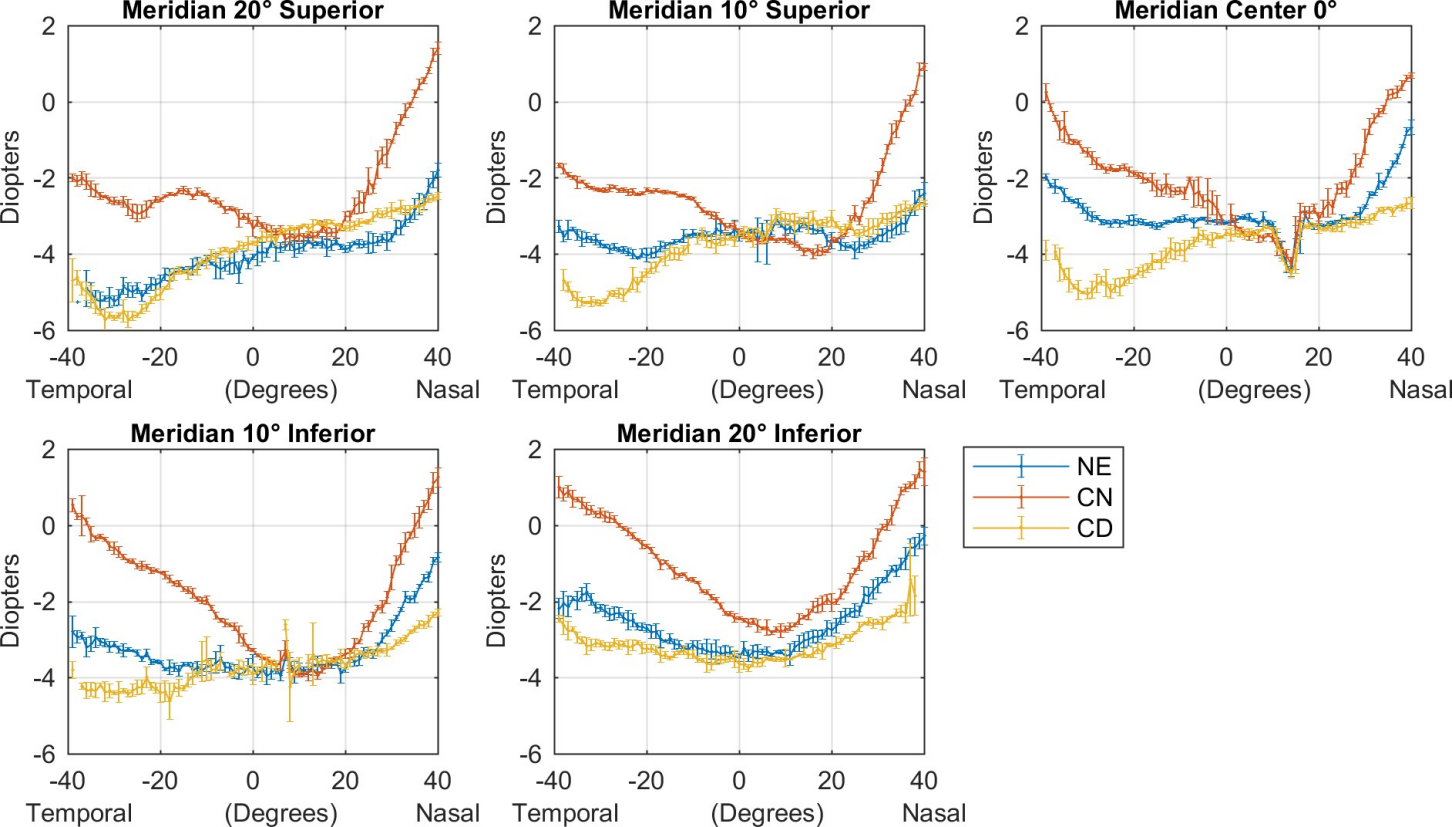
Error-bar plots of the peripheral refraction and “over-refraction” with the different contact lenses for one subject in each one of the five meridians in-use. In blue, peripheral refraction or naked eye; in red, over-refraction with near-centre design contact lens; in yellow, over-refraction with distance-centre refraction.

All baseline conditions were analysed separately for the real measured points and the interpolated matrices before mixing them. The analysis of the real measured values of the peripheral refractive error, show differences between each group (p>0.05, Kruskal-Wallis with false discovery rate (FDR)). Because of the post-measured filter, the final standard deviation of all those measured points was ±0.26 D. Fig 2, shows the peripheral refractive measurements for the same subject with each one of the three optical conditions (naked eye, centre-near lens and centre-distance lens).

**Fig 3:**
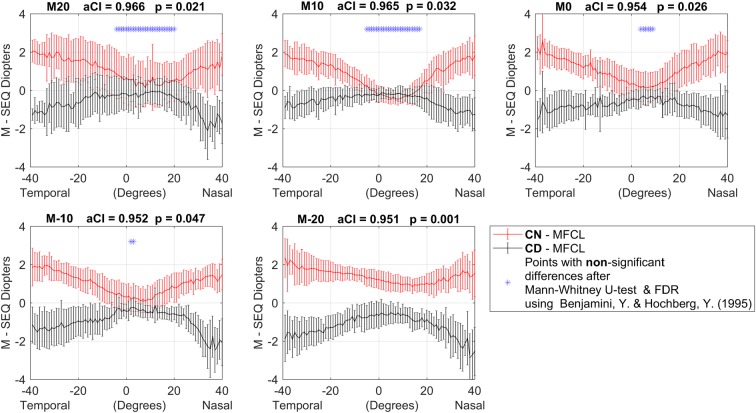
Average and SD of the SE contact lens profiles, after subtracting the naked eye conditions (n = 13). Images from top to bottom and left to right represent the different meridians measured. Y-axis: dioptres scale; X-axis: degrees scale. The points notated with a blue ‘*’ are the points where non-significant statistical differences were found, after applying Mann-Whitney-U-test and the Benjamini-Hochberg FDR correction.

Similarly, the results of the measured points in the contact lens power profiles (as the average from the dioptric difference between the naked eye peripheral refraction and the ‘over-refraction’ with the contact lenses) were found to be significantly different using the non- parametric T-test, Mann-Withney-U, with Benjamini-Hochberg FDR defining the new critical p-value for each meridian (always below 0.05). Detailed results are plotted in each meridian and can be observed along with their final critical p-value in Fig 3, where some non-significant differences can be appreciated in the superior and central parallels.

In terms of the full-field interpolated matrices, the original maps from the scenes were found to be statistically different (p < 0.05, Kruskal-Wallis with false discovery rate (FDR)) in all the regions. Multiple pairwise comparisons agreed on all, but the adjoining temporal region (from 15 to 20°) between scene 2 (Corridor) and scene 3 (Living Room).

Along with the results from the environment, the interpolated matrices of the proposed types of peripheral refractive errors differed in all the defined regions (p < 0.05, Kruskal-Wallis with FWER). Contrary to the original measured points where some points showed no differences in the central degrees and especially in the superior parallels (10° and 20°), the full-field matrices from the contact lenses revealed that different lens designs behave differently in all the tested regions (p < 0.05, Mann-Whitney-U with FDR).

In summary, it can be concluded that the baseline conditions matrices were statistically different each other before they were combined. Therefore, non-significant differences can be mostly owned to the combination of the conditions rather than the individual original conditions.

### Defocus patterns maps

The final merged maps were compared using the non-parametric Kruskal-Wallis test at the 0.05% significance level. Multiple pairwise comparisons with familywise error algorithms were applied afterwards to all the merged map´s areas, and statistically significant differences were defined by a critical p-value < 0.0326, after the correction.

The outermost inferior region, from 15 to 20°, was found to be statistically different at all 36 conditions tested. By taking the superior region into account, the outermost region (15 to 20°) was significantly different for all conditions tested. Only in case of Scene 2 in combination with the centre-near design of the contact lens and the TPS refractive profile, no significant differences were found. In the remaining areas, although significant changes appeared, some consistent exceptions were found. For example, no differences were found for the nasal regions 0 to 10° between NPS and RPH, no matter which optical solution was worn or which scene was analysed. On behalf of the contact lens designs, the centre-near design was unable to produce significant changes within the Inferior and Nasal, 5° to 15° regions, in those cases when the subject had a TPS refraction profile. Additionally, that contact lens design was also not capable of changing the Inferior (10° to 15°) region for the subjects with an NPS profile. Lastly, the highest number of non-statistical differences was found in the temporal segment between scene 2 (Corridor) and 3 (Living Room), which is in line with the baseline results. The scenes were also unable to promote statistically significant changes in the Nasal region of 10° to 20° and the temporal regions (5° to 20°).

**Fig 4:**
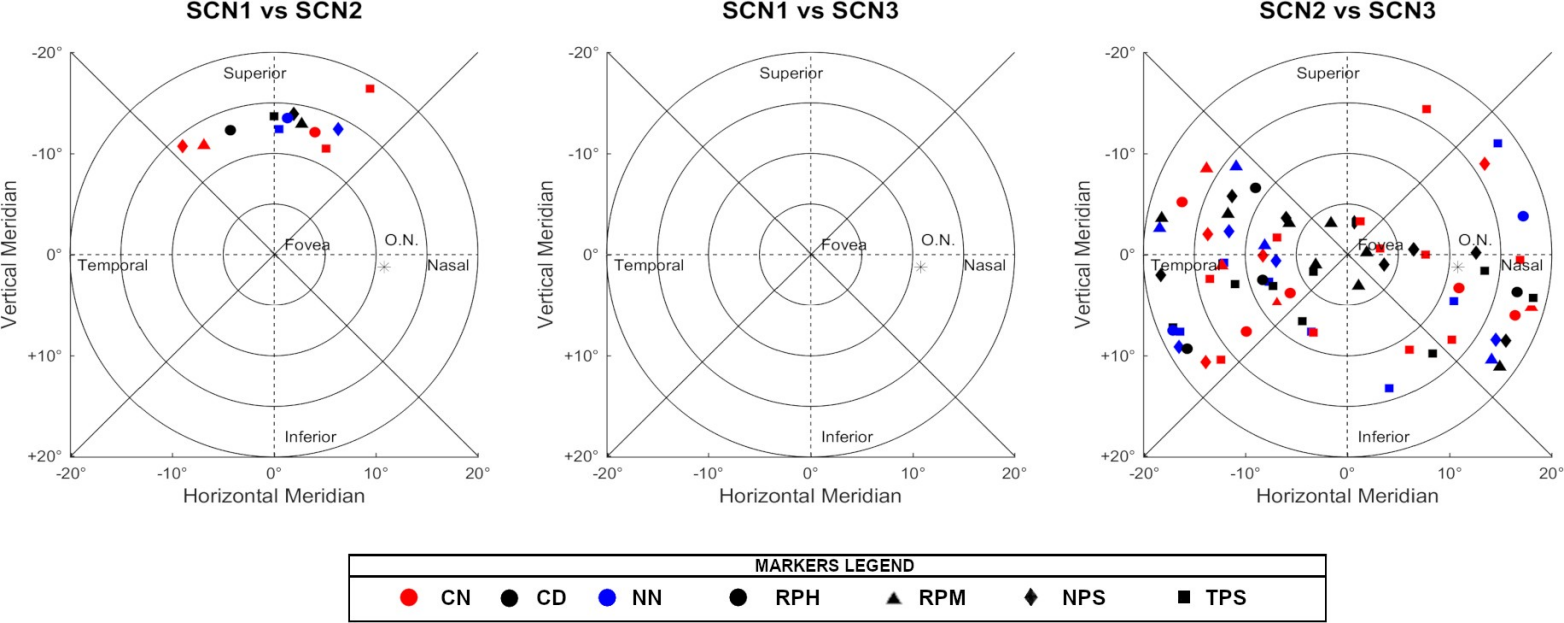
Non-significant differences in the post-hoc analysis when comparing the different scenarios. The absence of markers indicates that those regions were significantly different under all conditions tested.

**Fig 5:**
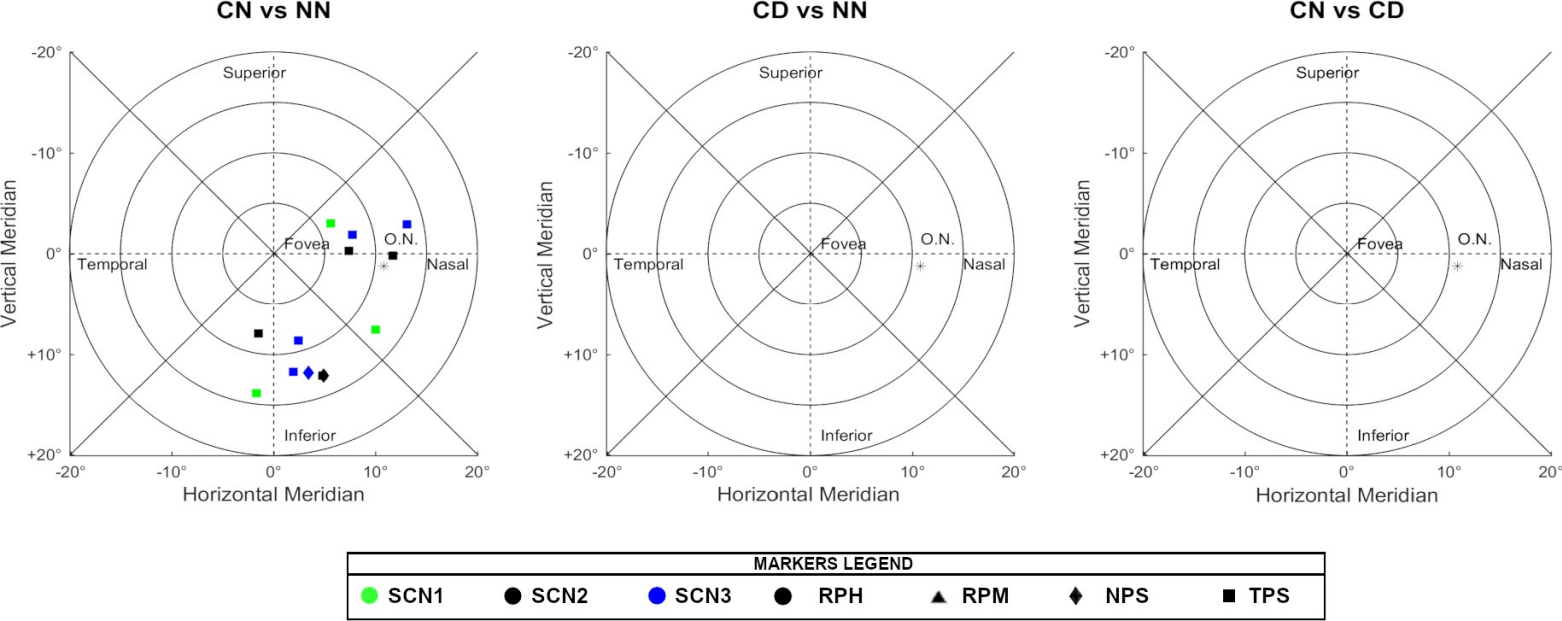
Non-significant differences in the post-hoc analysis when comparing the different optical treatments. The absence of markers indicates that those regions were significantly different under all conditions tested.

**Fig 6:**
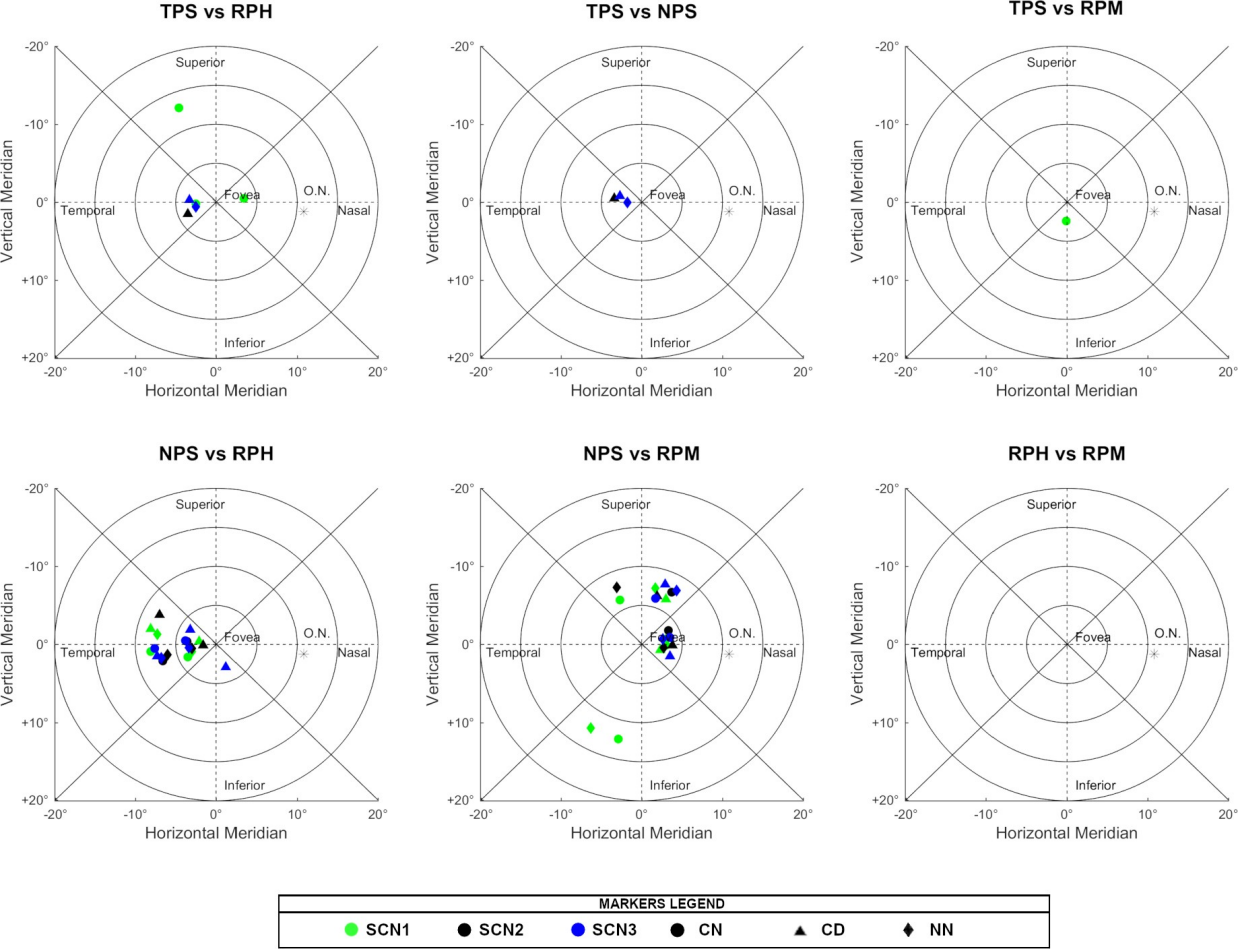
Non-significant differences in the post-hoc analysis when comparing the different types of peripheral refraction. The absence of markers indicates that those regions were significantly different under all conditions tested.

In the following Figs 4–6, there are all the non-significant differences addressed in groups after the post-hoc analysis.

### Asymmetries on the measured contact lens profiles

**Fig 7:**
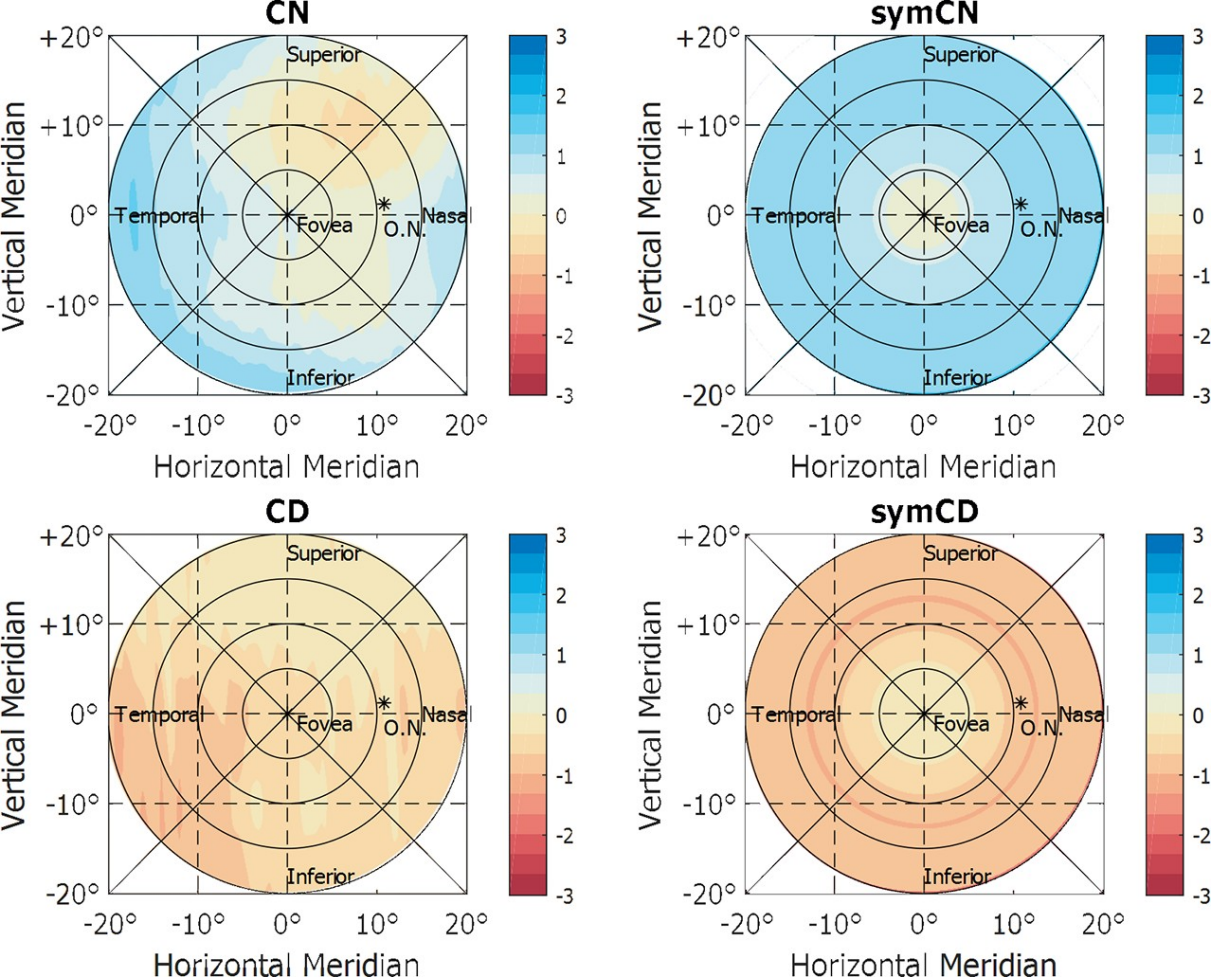
Computed, theoretical symmetric power profile of contact lenses, based on the values found in the measurements and the theoretical design of the contact lenses.

Another interesting finding was the fact that the measured contact lens profiles shown asymmetries within all subjects measurements, which is in disagreement with the proposed optical designs for myopia control [23]. Some asymmetries could be expected, as the general parameters of the contact lenses that were used during the study would not fit equally for the different eyes of the subjects. However, in this case, it should remain individually tied rather than being consecutively happening to all the subjects. For analysis, such possible behaviour in detail, theoretical models of symmetric designs were computed based on the measured power profiles, and the results are displayed in Fig 7.

These theoretical symmetric profiles were evaluated in the same manner than the original measurements, using the Kruskal-Wallis test and multiple pairwise comparisons with FWER. As a result, it was observed that symmetric profiles promoted significant changes for some regions where non-symmetrical ones did not, ending up with significant differences in all the regions tested for the lens conditions. On the other hand, the inherent differences to the peripheral refractive errors did not change, and some non-significant changes persisted for the skewed peripheral patterns.

## Discussion

Near-sightedness is a complex condition, where multiple factors have to be taken into account. The present study covers the information on all the different factors that constitute the blur signal at the retina for the first time. From environmental factors to the refractive shell, passing through the optical treatments, the optical system modifies the focal plane of the image that reaches each point of the retina.

Extensive literature can be found on the peripheral refractive errors (hereditary defocus due to dioptric uniformity) in myopic subjects, but almost none of the performed studies looked beyond the mean equator or horizontal meridian zero, being all self-limited to a single horizontal perspective[25,26]. In contrast, the current study provides rich details on peripheral refraction as this was measured for five different meridians.

The dioptric profiles (when described by the SE) from the contact lenses were completely unexpected, particularly the non-difference area in the centre where the addition power was supposed to be found [23,27]. The authors hypothesise that ‘over-refraction’ technique differs by measuring the effect of the contact lens on-wear rather than the absolute dioptric power. The aforementioned technique not only takes into consideration the tear layer but the placement of the contact lens on the eye. The unexpected profile might be acknowledged to several factors such as the possible role of accommodation, although measurements were performed while the subject fixated on a stimulus at three meters distance in order to avoid accommodation or perhaps owing to the fact that the profiles provided by the manufacturer did not correspond with the actual ones.

The overall obtained results from the matrices give us more information about the influence of soft multifocal contact lenses on retinal defocus patterns under real-life conditions, allowing better interpretation of the optical treatments that are currently used in myopia control.

From the results, it is clear that subjects with temporal or nasal positive skewed profiles of their peripheral refractive errors (TPS & NPS) are less receptive to significant changes that are caused by soft contact lenses with a near-centre design, compared to those subjects with relative peripheral myopic or hyperopic profiles (RPM & RPH), even when simulated symmetric contact lenses were used. If these results are replicated in clinical studies, they can serve as a reference for understanding why some subjects respond better than others do to optical treatments in controlling myopia. In congruence with our findings, the BHVI China Myopia Study reported that subjects presenting greater asymmetries on peripheral refraction were less prone to develop further myopia [28], which indicates that these profiles are also less receptive to environmental changes in defocus that induce myopia.

Both optical designs of the soft multifocal contact lenses were statistically different in the final maps, as it was expected. Given that the near-centre design promotes positive (myopic defocus) in the periphery and if the hypothesis that myopic defocus slow myopia progression is considered, we can indicate that the near-centre design used in this study might be adequate for myopia control. While this design was found to be unable to produce changes under a few different conditions for some of the regions, the conducted study also revealed statistically significant differences for most of the scenarios and regions. Nevertheless, it will be necessary to disclose whether these changes are also clinically meaningful.

Future studies will need to clarify several questions, for instance, which visual regions aside from the fovea matters most for myopia control [11], whether the peripheral defocus affect the onset or the progression of myopia or how the defocus is translated into eye growth. The literature also describes, how sensitivity to blur decreases with increasing distance from the fovea [11,29,30], suggesting that peripheral defocus may require greater magnitudes to result in detectable blur. Still, just as with accommodation, which is capable of being stimulated at much lower levels than the depth of focus (DOF) [31], the amounts of peripheral defocus may not necessarily need to be bigger than the DOF to trigger the myopia progression. Moreover, the blur sensitivity usually describes the ability to perceive the focus of the image, which additionally requires neural processing. Instead, it seems that the feedback loop for emmetropization occurs entirely in the retina[32] and perhaps due to some specific type of ganglion cells [33,34].

Certainly, the study brought on some limitations that can restrict its extent, for example, from the recording of the scenarios as already described in the original article[20]. The measurements of peripheral and contact lens profiles may have expanded those limitations. Namely, the number of subjects may have limited the definition of peripheral refractive profiles since, for example, the temporal skew profile was only found on a single subject. Therefore, the reliability of the temporal positive skew pattern may be reduced, although this pattern represents a less prevalent condition[26,35,36]. Furthermore, only two contact lenses were tested, and lastly, despite the behaviour was similar for the thirteen subjects, the profile of the contact lens might have been affected by the displacement of the same in the eye, because of the general fitting parameters used, the re-positioning of the lens after blinks or the cyclotorsion of the eye when shifting the gaze upwards or downwards.

## Conclusions

Despite the limited number of subjects, as well as the restricted number of scenarios and optical designs, given the ample amount of possible conditions, this study achieved several steps forward. With the presented research, it is possible to simulate how the blurring of the periphery changes or does not change under different optical designs /optical treatments for different subjects, and in different environments. The observed differences due to the type of peripheral refraction may explain the inter-subject variability in the progression of myopia that is typically observed in clinical trials. How exactly the retina detects the level of defocus is still a gap of knowledge. However, this work provides the ability to report the amounts of defocus under different conditions of optical correction or for different scenes, as well as for any area of the retina. Henceforth, a framework can be established giving the ability to test different optical designs even before going to clinical trials.
